# Structures of the APC–ARM domain in complexes with discrete Amer1/WTX fragments reveal that it uses a consensus mode to recognize its binding partners

**DOI:** 10.1038/celldisc.2015.16

**Published:** 2015-07-14

**Authors:** Zhenyi Zhang, Senem Akyildiz, Yafei Xiao, Zhongchao Gai, Ying An, Jürgen Behrens, Geng Wu

**Affiliations:** 1 School of Life Sciences and Biotechnology, State Key Laboratory of Microbial Metabolism, Shanghai Jiao Tong University, Shanghai, China; 2 Nikolaus-Fiebiger-Center for Molecular Medicine, University Erlangen-Nuremberg, Erlangen, Germany

**Keywords:** APC, Amer1, WTX, ARM domain, consensus recognition mode, crystal structure

## Abstract

The tumor suppressor APC employs its conserved armadillo repeat (ARM) domain to recognize many of its binding partners, including Amer1/WTX, which is mutated in Wilms' tumor and bone overgrowth syndrome. The APC–Amer1 complex has important roles in regulating Wnt signaling and cell adhesion. Three sites A1, A2, and A3 of Amer1 have been reported to mediate its interaction with APC-ARM. In this study, crystal structures of APC–ARM in complexes with Amer1-A1, -A2, and -A4, which is newly identified in this work, were determined. Combined with our GST pull-down, yeast two-hybrid, and isothermal titration calorimetry (ITC) assay results using mutants of APC and Amer1 interface residues, our structures demonstrate that Amer1-A1, -A2, and -A4, as well as other APC-binding proteins such as Asef and Sam68, all employ a common recognition pattern to associate with APC–ARM. In contrast, Amer1-A3 binds to the C-terminal side of APC–ARM through a bipartite interaction mode. Composite mutations on either APC or Amer1 disrupting all four interfaces abrogated their association in cultured cells and impaired the membrane recruitment of APC by Amer1. Our study thus comprehensively elucidated the recognition mechanism between APC and Amer1, and revealed a consensus recognition sequence employed by various APC–ARM binding partners.

## Introduction

Adenomatous polyposis coli (APC) is an important human tumor suppressor protein which plays critical roles in diverse cellular processes such as Wnt/β-catenin signaling [1], cell adhesion [2, 3], cell migration [4, 5], mitosis and chromosomal instability [6], and so on. Mutations of the human *APC* gene are found in most of familial adenomatous polyposis (FAP) patients, as well as in a majority of sporadic colorectal cancer cases [7, 8]. The N-terminal armadillo repeat (ARM) domain of APC is the most conserved region among its vertebrate and invertebrate homologs [9, 10], and mediates its association with a variety of binding partners including APC membrane recruitment 1 (Amer1, also named as WTX for ‘Wilms tumor gene on the X chromosome’) [11, 12], Asef [13], Sam68 [14], and IQGAP1 [15].

Amer1/WTX is another important human tumor suppressor whose gene is somatically inactivated in one-third of Wilms' tumors, the most common pediatric kidney cancers [12]. In contrast, germline mutations of the *Amer1/WTX* gene predispose to osteopathia striata congenita with cranial sclerosis, a bone overgrowth syndrome [16]. *Amer1/WTX*-knockout mice exhibited both kidney and bone defects due to aberrant specification of mesenchymal progenitor cell fate [17]. The Amer1 protein possesses two membrane targeting regions at its N-terminus, three APC-interacting sites named as A1 (residues 280–368), A2 (residues 380–531), and A3 (residues 717–834) [11], as well as a β-catenin-binding arginine-glutamate-alanine (REA) repeats [18] (Supplementary Figure S1). By recruiting APC to the plasma membrane, Amer1 regulates the APC-dependent maintenance of intercellular junctions [11]. In addition, together with APC and Axin, Amer1 promotes ubiquitination and degradation of β-catenin, thus negatively regulates the Wnt signal transduction pathway [19]. Moreover, Amer1 also has a variety of other functions including participating in the upstream activation of Wnt signaling by promoting the phosphorylation of the Wnt co-receptor LRP6 [20], shuttling to the nucleus and enhancing the transcriptional activity of Wilms tumor 1 (WT1) [21], and positively regulating the CBP/p300-mediated p53 acetylation [22].

The three APC-binding regions A1, A2, and A3 of Amer1 display no obvious sequence similarities among themselves nor exhibit any resemblance with other APC-ARM-binding motifs such as the APC-binding region (ABR) of Asef [10] and the YY motif of Sam68 [14]. These three sites have only been roughly mapped to regions spanning 50–120 residues, and it is completely unknown which residues at these three sites mediate specific recognitions with APC. In the case of the ARM domain of β-catenin, its interaction partners such as phospho-APC 20-amino-acid repeat [23, 24], phospho-E-cadherin cytoplasmic domain [25], and TCF’s β-catenin-binding domain [26] all reside in the same surface groove of β-catenin-ARM with a similar binding pattern despite their diverse sequences. It will be interesting to examine whether different APC–ARM-binding fragments including those from Amer1, Asef, and Sam68 also exhibit a common mode to associate with APC-ARM, although they have no apparent recognizable homology with each other.

In this study, we investigated the molecular basis underlying the complex formation between Amer1 and APC. We identified core sequences of the Amer1-A1 and -A2 sites for APC binding, discovered a fourth APC-binding site on Amer1 (which we named as A4), and determined the crystal structures of APC–ARM in complexes with the A1, A2, and A4 peptides of Amer1. In addition, point mutations of key interface residues on Amer1-A1/A2/A4 or APC–ARM abrogated their interactions by glutathione sulfur transferase (GST) pull-down, yeast two-hybrid, and isothermal titration calorimetry (ITC) assays. In contrast to Amer1-A1/A2/A4, Amer1-A3 was found to use a bipartite interaction mode to bind to the C-terminal side of APC–ARM. Composite mutations on APC or Amer1 disrupting all four interaction interfaces abolished their association in cultured cells and abrogated the membrane recruitment of APC by Amer1. Finally, structural superimposition reveals a common mode used by APC–ARM to recognize Amer1-A1/A2/A4, Asef-ABR, and Sam68-YY. By summarizing their sequences and interaction patterns, a consensus APC–ARM recognition motif employed by its binding partners is proposed.

## Results

### Identification of the core Amer1-A1 and -A2 sequences for APC–ARM binding

**Figure 1:**
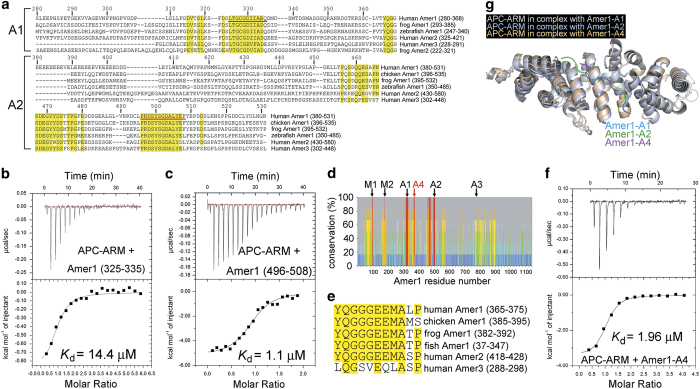
Identification of a fourth APC-binding fragment A4 of Amer1/WTX, and crystal structures of APC–ARM in complex with the A1, A2, and A4 fragments of Amer1. (**a**) Sequence alignment of the APC-binding A1 and A2 fragments of human Amer1 (hAmer1). hAmer1-A1 (residues 280–368) and -A2 (residues 380–531) are aligned with orthologs and paralogs. The A1 and A2 fragments used in the crystallization experiments are marked with red underlines. Residues identical in all the Amer1 homologs are highlighted in yellow. Residue numbers for hAmer1 are indicated above the sequences. (**b**, **c**) The binding affinities of hAmer1 (residues 325–335, (**b**) and hAmer1 (residues 496–508, (**c**) for APC–ARM. (**d**) Sequence comparison of human, chicken, frog, and zebrafish Amer1, human Amer2 and Amer3 reveals a highly conserved fragment, which is named as A4. hAmer1 residue numbers are indicated, and the percentage of conservation for each residue is shown as a red, orange, yellow, green, cyan, and blue bar from high to low conservation, respectively. The membrane-binding regions M1 and M2, as well as the other APC-binding sites A1, A2, and A3, are marked. (**e**) Sequence alignment of the A4 site (residues 365–375) of hAmer1 and its homologs. Residues identical in at least 5 out of 6 homologs are highlighted in yellow. (**f**) The dissociation constant (*K*_d_) between APC–ARM and Amer1-A4 was measured to be 1.96 μM by the ITC assay. (**g**) Overall crystal structures of APC–ARM in complexes with hAmer1-A1 (325–335), -A2 (496–508), and -A4 (365–375).

Previous studies have roughly mapped the Amer1-A1 and -A2 sites to residues 280–368 and 380–531, respectively [11]. However, examination of the structure of APC–ARM shows that the ligand-binding groove on its surface is ~50 Å long and~20 Å wide [9, 10], which can only accommodate a peptide of 12–15 residues in a fully extended conformation or a longer peptide containing bends or secondary structures. Alignment of the human Amer1 protein sequence with those of its chicken, frog, and zebrafish orthologs as well as its paralogs Amer2/FAM123A and Amer3/FAM123C revealed conserved sequence blocks (Amer1 residues 315–335 and 496–508 for the A1 and A2 sites, respectively) flanked by non-conserved residues (Figure 1a) [27]. Intriguingly, ITC experiments demonstrated that peptides of Amer1-A1 (residues 325–335) and -A2 (residues 496–508) sequences exhibited approximately equal binding affinities for APC–ARM (Figures 1b and c, Table 1) as full Amer1-A1 (residues 280–368) and -A2 (residues 380–531), respectively (Table 1, Supplementary Figures S2A and S2B). In previous studies, the N507K point mutation of APC has been shown to abrogate its association with Amer1 [11] and Asef [10, 28], and its corresponding mutation of N175K in *Drosophila* APC2 (also known as E-APC) resulted in developmental defects [2]. In accordance with these findings, both Amer1 (325–335) and Amer1 (496–508) displayed non-detectable interaction with the N507K point mutant protein of APC–ARM, as shown by the GST pull-down assay (Supplementary Figure S3). Therefore, Amer1 residues 325–335 and 496–508 are regarded to represent the core A1 and A2 fragments for APC binding, respectively, although other conserved Amer1 residues nearby such as residues 315–324 may also contribute to the interaction with APC.

### Identification of A4, a fourth APC-binding site on Amer1

Amino-acid sequences of the three APC-binding sites of Amer1/WTX, especially A1 and A2, show little variance among different Amer1 orthologs and paralogs (Figures 1a and 1d), suggesting that the recognition of APC is a conserved function of Amer1 throughout evolution. When the percentage of conservation for each Amer1 residue was carefully examined, another highly invariant sequence block between the A1 and A2 sites, residues 365–375, was found (Figures 1d and e) [27]. Similar to the core A1 and A2 sequences, it also contains an acidic residue in the middle and several hydrophobic residues at its C-terminal part (Figure 1e). When the ITC assay was performed to examine the binding affinity between Amer1 (365–375) and APC–ARM, their dissociation constant (*K*_d_) was measured to be 1.96 μM (Figure 1f), stronger than that of Amer1-A1 and similar to that of Amer1-A2 (Figures 1b and c). Hence, this sequence block represents a fourth motif on Amer1 for APC recognition, which is named as A4 after the three previously described APC-binding sites.

### Crystal structures of the APC–ARM/Amer1-A1, APC–ARM/Amer1-A2, and APC–ARM/Amer1-A4 complexes

To further understand the molecular basis of how APC recognizes Amer1, we determined the crystal structures of the APC–ARM/Amer1-A1, APC–ARM/Amer1-A2, and APC–ARM/Amer1-A4 complexes to 1.90 Å, 2.00 Å, and 1.70 Å resolutions, respectively (Table 2). Despite possessing apparently different sequences, Amer1-A1, -A2, and -A4 adopt remarkably similar conformations when bound to APC–ARM, both in anti-parallel manners with respect to the armadillo repeats of APC (Figure 1g).

**Figure 2:**
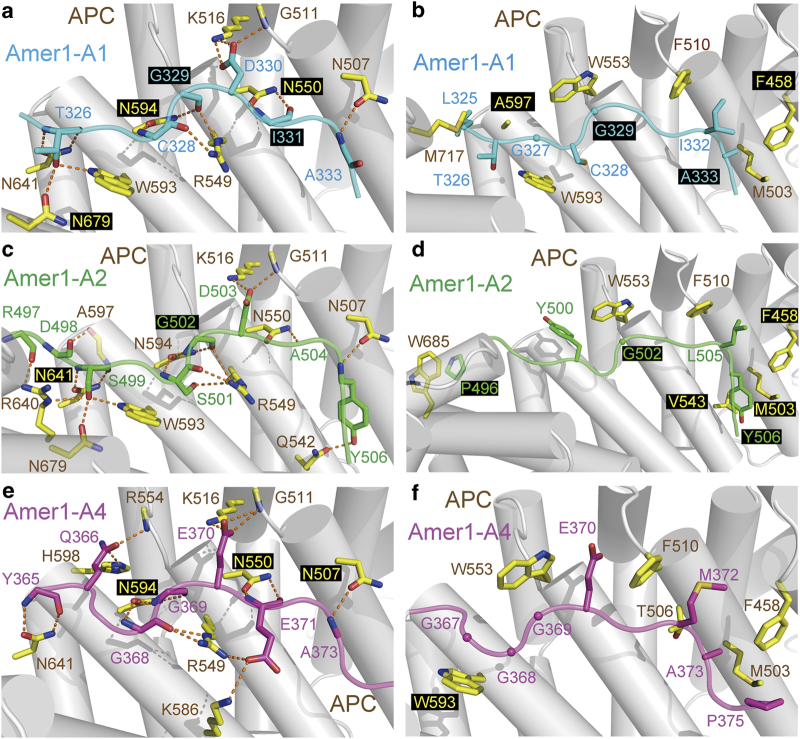
Interaction interfaces of Amer1-A1, -A2, and -A4 with APC–ARM. (**a**, **b**) Hydrogen bonding (**a**) and van der Waals interactions (**b**) between APC–ARM and Amer1-A1. (**c**, **d**) Hydrogen bonding (**c**) and van der Waals interactions (**d**) between APC–ARM and Amer1-A2. (**e**, **f**) Hydrogen bonding (**e**) and van der Waals interactions (**f**) between APC–ARM and Amer1-A4. Nitrogen and oxygen atoms are colored in blue and red, respectively. Carbon atoms of Amer1-A1, -A2, -A4, and APC–ARM are shown in cyan, green, magenta, and yellow, respectively. Hydrogen bonds are indicated as orange dashed lines.

At all three interfaces between APC–ARM and Amer1-A1/A2/A4, four highly conserved asparagine residues, N507/N550/N594/N641 from the H3 helices of armadillo repeats 2/3/4/5 of APC–ARM function as rivets fastening Amer1-A1/A2/A4 onto its surface groove. With the exception of N507 that forms only one hydrogen bond with A1-A333/A2-A504/A4-A373, these asparagines each employs its side chain amide group to make a couple of hydrogen bonds with the main chain NH and CO groups of Amer1-A1, -A2, and -A4 peptides (Figures 2a, c and e). In addition, two basic residues, APC-K516 and -R549, straddle the middle portion of Amer1-A1/A2/A4 peptides on both sides. They form salt bridges with a conserved acidic residue A1-D330/A2-D503/A4-E370 and make hydrogen bonds with the main chain carbonyl groups of A1-C328/A1-G329/A2-S501/A2-G502/A4-G368 (Figures 2a, c and e). Moreover, two tryptophan residues, APC-W593 and -W553, provide more binding affinity by hydrogen bonding with A1-T326/A2-S499 and making van der Waals interactions with A1-G327/A1-G329/A2-Y500/A2-G502/A4-G367/A4-G369, respectively (Figures 2b, d and f). Furthermore, a number of hydrophobic residues F458, M503, and F510 from APC cluster together and form hydrophobic contacts with non-polar residues such as I332/A333 from Amer1-A1, L505/T506 from Amer1-A2, and M372/A373 from Amer1-A4 (Figures 2b, d and f). Therefore, Amer1-A1, -A2, and -A4 all orchestrate a remarkably similar assembly of hydrogen bonds and van der Waals interactions to associate with an almost identical set of APC–ARM surface groove residues, despite their apparently divergent sequences.

### Mutations of key residues of APC–ARM and Amer1-A1/A2/A4 disrupt their associations

To corroborate our structural observations, we performed site-directed mutagenesis on critical interface residues of APC–ARM and Amer1-A1/A2/A4, and examined the effects of these point mutations by yeast two-hybrid, GST pull-down, and ITC assays.

**Figure 3:**
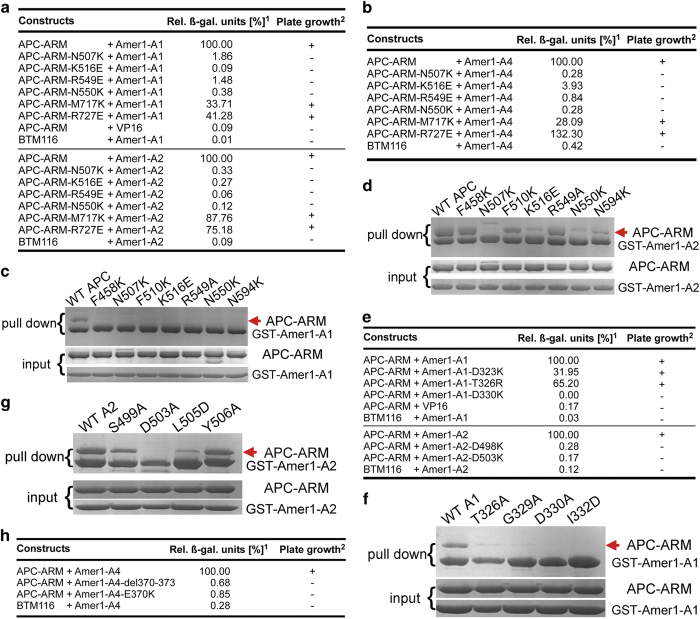
Mutation of critical APC or Amer1-A1/A2/A4 interfaces residues abrogated the associations of APC–ARM with Amer1-A1, -A2, and -A4. (**a**, **b**) Point mutants of crucial interface residues on APC–ARM were defective in recognizing murine Amer1-A1 (residues 279–367) (**a**), -A2 (residues 388–551) (**a**), and hAmer1-A4 (residues 337–455) (**b**) in the yeast two-hybrid assay. ^1^ Relative β-galactosidase reporter activity. ^2^ Growth of transformed yeast on –His selective media. Expression levels of WT and mutant APC–ARM constructs used are shown in Supplementary Figure S4. (**c**, **d**) Substitutions of key APC–ARM residues abolished its complex formation with hAmer1-A1 (**c**) and -A2 (**d**), as demonstrated by the GST pull-down assay. (**e**) Point mutations of key interface residues on hAmer1-A1 (residues 280–369) or -A2 (residues 380–531) disrupted their interactions with APC–ARM, as shown by the yeast two-hybrid assay. Expression levels of WT and mutant Amer1-A1/A2 constructs used are shown in Supplementary Figure S6. (**f**, **g**) hAmer1-A1 (**f**) and -A2 (**g**) mutants with key APC–ARM-interacting residues altered had diminished affinities for APC–ARM, as revealed by the GST pull-down assay. (**h**) Point mutation or deletion of key interface residues on hAmer1-A4 (residues 337–455) abolished its interaction with APC–ARM, as demonstrated by the yeast two-hybrid assay. Expression levels of WT and mutant hAmer1-A4 constructs used are shown in Supplementary Figure S8.

Consistent with their crucial roles observed in the co-crystal structures, replacing the three key asparagines of APC, N507/N550/N594, by lysines disrupted or diminished the association between APC–ARM and Amer1-A1/A2/A4 (Figures 3a–d, Supplementary Figure S4). In addition, point mutations of K516E and R549E (and to a less extent, R549A) abolished the recognition of APC–ARM for Amer1-A1/A2/A4 (Figures 3a–d, Supplementary Figures S4 and S5). Furthermore, substitution of the crucial hydrophobic residue APC-F510 by a lysine reduced the complex formation between APC–ARM and Amer1-A1 (Figure 3c).

In Amer1-A1, -A2, and -A4, a serine/threonine residue (A1-T326/A2-S499), an acidic residue (A1-D330/A2-D503/A4-E370), and a non-polar residue (A1-I332/A2-L505/A4-M372) are key contributors mediating interactions with APC–ARM (Figure 2). Accordingly, point mutations of T326R/T326A, D330K/D330A, and I332D in Amer1-A1 eliminated its binding with APC–ARM (Figures 3e and f, Supplementary Figure S6); the S499A, D503K/D503A, and L505D mutations in Amer1-A2 undermined its complex formation with APC–ARM (Figures 3e and g, Supplementary Figures S6 and S7); and the E370K point mutation or deletion of residues 370–373 in Amer1-A4 eliminated its interaction with APC–ARM (Figure 3h, Supplementary Figure S8). Moreover, the G329A mutation in Amer1-A1 (Figure 3f) and the D498K mutation in Amer1-A2 (Figure 3e) also attenuated their associations with APC–ARM as well.

### Different from Amer1-A1/A2/A4, Amer1-A3 employs a bipartite binding mode to interact with the C-terminal side of APC–ARM

**Figure 4:**
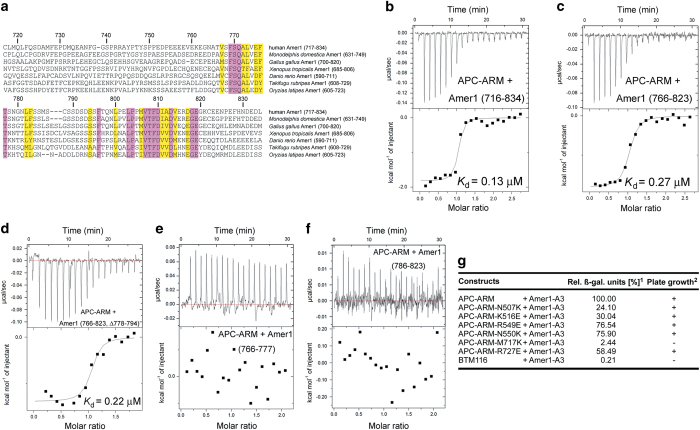
Different from A1/A2/A4, Amer1-A3 employs a bipartite binding mode to associate with the C-terminal side of APC–ARM. (**a**) Sequence alignment of the A3 regions of different Amer1 homologs. Residues identical/similar in all seven Amer1 homologs analyzed here are highlighted in pink/yellow, respectively. (**b**–**f**) Identification of the minimal A3 fragment for APC binding. Binding affinities of Amer1 (716–834) (**b**), Amer1 (766–823) (**c**), and Amer1 (766–823, Δ778–794) (**d**) for APC–ARM are all approximately similar, as measured by the ITC assay. In contrast, Amer1 (766–777) (**e**) and Amer1 (786–823) (**f**) have no detectable binding affinities for APC–ARM. (**g**) Point mutation of M717K at the C-terminal side of APC–ARM eliminated its association with Amer1-A3 (murine, residues 721–838), as revealed by the yeast two-hybrid assay. Point mutation of N507K, K516E, R549E, and N550K on APC–ARM did not affect its interaction with Amer1-A3. ^1^ Relative β-galactosidase reporter activity. ^2^ Growth of transformed yeast on –His selective media.

The APC–ARM-binding site A3 of Amer1 was only roughly mapped to a region of about 120-amino acids from 717 to 834 [11]. Sequence comparison of the human Amer1-A3 with its orthologs shows that residues 767–819 are highly conserved, whereas residues outside this region possess little homology (Figure 4a). Indeed, when we compared the binding of APC–ARM with Amer1 (716–834) or Amer1 (766–823) by the ITC and GST pull-down assays, these two constructs of Amer1 exhibited comparable interaction affinities (Figures 4b and c, Table 1, Supplementary Figure S9A), suggesting that Amer1 (residues 766–823) might represent the core A3 fragment for associating with APC.

Within Amer1 (766–823), residues 767–777 and 796–819 are more conserved, while the amino acids 778–795 between them display much more diversity (Figure 4a). When residues 778–794 were deleted from Amer1 (766–823), the resulting construct Amer1 (766–823, Δ778–794) still retained the majority of binding affinity for APC–ARM (Figures 4c and d, Table 1, Supplementary Figure S9A). To exclude the possibility that either the N-terminal (residues 766–777) or C-terminal fragment (residues 795–823) of Amer1 (766–823, Δ778–794) was sufficient for binding to APC–ARM, we examined the association of Amer1 (766–777) or Amer1 (786–823) with APC–ARM by the GST pull-down (Supplementary Figure S9B) and ITC assays (Figures 4e and f, Table 1). Our results showed that Amer1 (766–777) or Amer1 (786–823) displayed no detectable interactions with APC–ARM. Residues 778–794 probably form a bulging loop when Amer1-A3 is bound to APC–ARM, and thus do not provide much contribution to the recognition between APC and Amer1. Therefore, Amer1-A3 employs residues 766–777 and 795–823 to associate with APC–ARM by a bipartite binding mechanism, and neither of these two fragments is able to interact with APC–ARM separately on its own.

When we tested the set of APC–ARM point mutants defective in binding Amer1-A1/A2/A4 such as N507K/K516E/R549E/N550K for association with Amer1-A3, we surprisingly found that these mutations did not affect their interaction (Figure 4g). In contrast, a point mutation at the C-terminal side of APC–ARM, M717K, destroyed the APC–ARM/Amer1-A3 complex formation (Figure 4g). M717 was mutated because it was shown to be a key residue in mediating the interaction of APC–ARM with Sam68 [14] and lies outside of the residues mediating interaction of APC–ARM with Amer1-A1/A2/A4, which were not relevant for binding to Amer1-A3. Hence, Amer1-A3 interacts with the C-terminal portion of APC–ARM using a bipartite binding mode, which is different from the way that Amer1-A1/A2/A4 bind.

### Composite mutations on Amer1 (2–700) disrupting the A1/A2/A4 sites abrogated its association with FL APC and compromised the recruitment of APC to the plasma membrane

**Figure 5:**
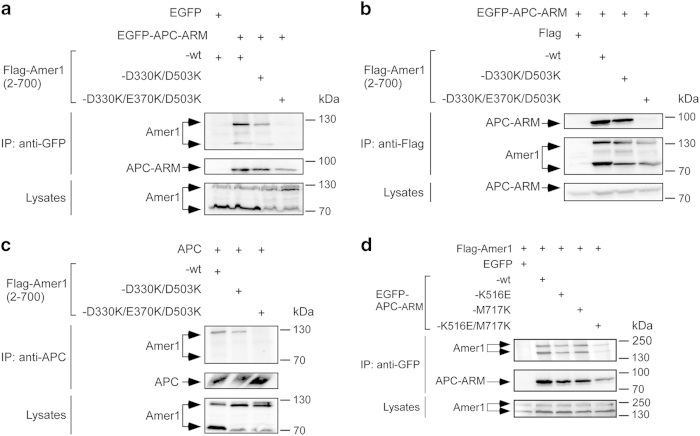
Composite mutations in Amer1 or APC abolished the Amer1–APC interaction in cultured cells. (**a**, **b**) Co-immunoprecipitation assays demonstrated that triple mutation of A1-D330K/A4-E370K/A2-D503K in Flag-tagged Amer1 (residues 2–700) attenuated its association with EGFP-tagged APC–ARM, using either anti-GFP (**a**) or anti-Flag (**b**) immunoprecitation followed by western blot analysis. Note that transfection of the Amer1 cDNA generates two protein bands that are due to alternative splicing (arrows [18]). (**c**) Triple mutation of D330K/E370K/D503K in Amer1 (2–700) destroyed its interaction with FL APC. (**d**) Double mutation of K516E (which abrogated A1/A2/A4-binding) and M717K (which impaired A3-binding) in APC–ARM reduced its association with FL Amer1 by 60% as determined by densitometric analysis.

Having examined the interaction mechanism between APC–ARM and each of Amer1-A1/A2/A3/A4 sites, we next examined what the effects would be when mutations targeting different sites were combined. To this end, we first prepared a triple mutant construct of Amer1 (2–700) in which the A1/A2/A4 sites were destroyed by the D330K/D503K/E370K triple mutation. Amer1 (2–700) does not contain the A3 site, thus this triple mutant construct effectively has all of the APC-binding sites removed. Indeed, co-immunoprecipitation assays verified that Amer1 (2-700, D330K/D503K/E370K) was not able to assemble with either APC–ARM (Figures 5a and b) or FL APC (Figure 5c) anymore, although the Amer1 (2–700) double mutant D330K/D503K with only the A1/A2 sites disrupted still associated with APC (Figures 5a and c).

**Figure 6:**
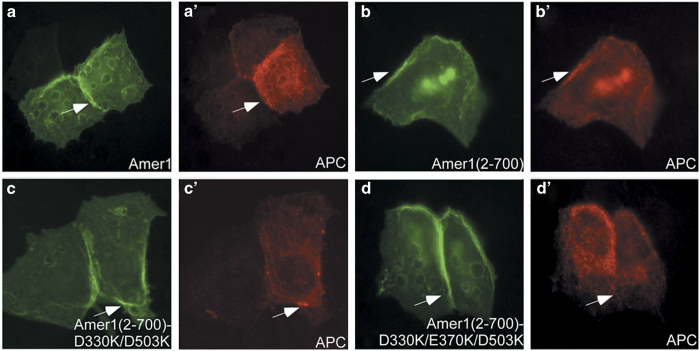
Amer1 (2–700) with critical A1/A2/A4 interface residues mutated was not able to recruit APC to the plasma membrane. EGFP-tagged FL Amer1 or WT/point mutants of Amer1 (2–700) as well as FL APC were transiently transfected into MCF7 cells, followed by immunofluorescence analysis. APC was detected using the antibody Ali. Arrows indicate the localization of Amer1 and APC. (**a**, **a**ʹ) FL Amer1 recruited APC to the plasma membrane. (**b**, **b**ʹ) WT Amer1 (2–700) also carried APC to the plasma membrane. (**c**, **c**ʹ) The A1/A2 sites double mutant D330K/D503K of Amer1 (2–700) still translocated APC to the plasma membrane. (**d**, **d**ʹ) The A1/A2/A4 sites triple mutant D330K/D503K/E370K of Amer1 (2–700) failed to recruit APC to the plasma membrane.

One important function of Amer1 is to recruit APC to the plasma membrane, thus regulating the formation of intercellular junctions and Wnt signaling (Figures 6a and aʹ) [11, 18]. Similarly, Amer1 (2–700) could also bring APC to the plasma membrane (Figures 6b and bʹ). When the double mutation of D330K/D503K with only the A1/A2 sites destroyed was introduced to Amer1 (2-700), APC was still recruited to the plasma membrane by Amer1 (Figures 6c and cʹ). In contrast, the D330K/D503K/E370K triple mutation on Amer1 (2–700), which eliminated all four APC-binding sites, abrogated the membrane co-localization of APC with Amer1 (Figures 6d and dʹ). Therefore, our co-immunoprecipitation and subcellular co-localization experiments demonstrate that each of the four APC-binding motifs of Amer1 is functional in recruiting APC in cultured cells and translocating APC to the plasma membrane.

### Composite mutations of APC–ARM destroying all four Amer1-binding interfaces reduced its complex formation with FL Amer1

As described above, the K516E mutation on APC–ARM impaired its association with Amer1-A1/A2/A4 (Figures 2a and b). In contrast, the M717K mutation on APC–ARM diminished its binding to Amer1-A3 (Figure 3g). However, single-point mutants of APC–ARM-K516E or -M717K were still co-immunoprecipitated with FL Amer1/WTX, presumably due to interactions mediated by the remaining binding interface(s) (Figure 5d). In contrast, the double mutation of K516E/M717K on APC–ARM strongly reduced its complex formation with FL Amer1 (Figure 5d). Therefore, disruption of all four binding interfaces A1/A2/A3/A4 of Amer1 is necessary and sufficient to dissociate its complex formation with APC.

### Structural comparison reveals that APC–ARM employs the same surface groove to recognize a consensus motif from Amer1-A1, -A2, -A4, Asef-ABR, and Sam68-YY

**Figure 7:**
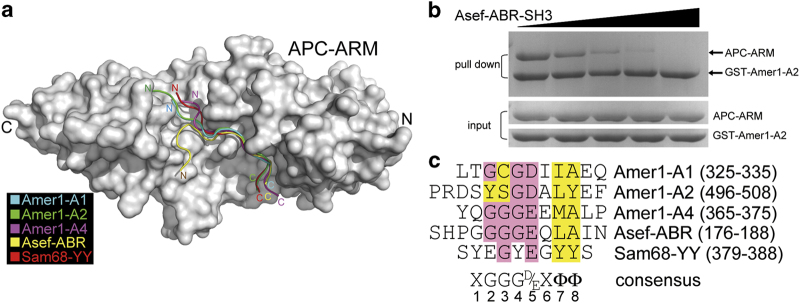
Comparison of the binding patterns of Amer1-A1, -A2, -A4, Asef-ABR, and Sam68-YY reveals a common recognition motif for APC–ARM association. (**a**) Structural superimposition of APC–ARM in complexes with its binding partners (Amer1-A1: cyan, Amer1-A2: green, Amer1-A4: magenta, Asef-ABR: yellow, and Sam68-YY: red). (**b**) Competition between Asef-ABR-SH3 and Amer1-A2 for binding to APC–ARM using the GST pull-down assay. (**c**) Structure-based alignment of the APC-binding sequences of human Amer1-A1, -A2, -A4, Asef-ABR, and Sam68-YY. The consensus APC–ARM binding motif XGGGD/EXΦΦ (X stands for any residue, and Φ represents a hydrophobic residue) is shown below the sequences.

Amer1-A1, A2, and A4 have no apparent sequence similarity among each other, nor do they bear any resemblance with the APC-binding regions of Asef and Sam68. Yet, by superimposing the crystal structures of APC–ARM in complexes with Amer1-A1/A2/A4 as well as Asef-ABR and Sam68-YY [10, 14], these APC-interacting ligands are all found to reside in the same surface groove of APC–ARM, all in anti-parallel manners with respect to the armadillo repeats of APC (Figure 7a). As these APC-binding partners occupy roughly the same position, we wonder whether they would compete with each other in a mutually exclusive manner. Indeed, addition of the Asef-ABR-SH3 protein to the pre-assembled APC–ARM/Amer1-A2 complex progressively dissociated APC–ARM from Amer1-A2 in a dose-dependent manner (Figure 7b), which is consistent with our surmise.

Although these APC-binding ligands look apparently very different at the first glance, a concealed consensus pattern XGGGD/EXΦΦ (X stands for any residue and Φ represents a hydrophobic residue) among these motifs was surprisingly found when we carefully compared their sequences (Figure 7c). The first residue varies among different APC-interacting motifs (T326 in Amer1-A1, S499 in Amer1-A2, Q366 in Amer1-A4, and Y380 in Sam68-YY). Its backbone is generally recognized by the formation of hydrogen bonds with the side chain amide group of APC-N641 (Q366 in Amer1-A4 is the exception), while its side chain normally forms polar contacts with APC-N679 and -W593 (Q366 in Amer1-A4 and Y380 in Sam68-YY are the exceptions).

The second, third, and fourth positions are generally glycines, which are contacted by the bulky side chains of W552 and W593, and hydrogen bonded with R549 and N594 of APC. Notably, the glycine residue at the fourth position of Amer-A1, -A2, and -A4 peptides (G529, G502, and G369, respectively) all adopt special dihedral angles in our crystal structures that enable them to access regions of the Ramachandran plot disallowed for the other nineteen kinds of amino acids (Supplementary Table S1). This is consistent with the fact that the fourth glycine is the most conserved one among the three glycine residues of the consensus motif (Figure 7c).

The fifth amino acid of the APC–ARM-binding consensus is an acidic one (D330 in Amer1-A1, D503 in Amer1-A2, E370 in Amer1-A4, E183 in Asef-ABR, and E384 in Sam68-YY), whose side chain carboxylate group is recognized by G511 and K516 of APC by hydrogen bonds and salt bridges. In contrast, the seventh and eighth sites of the consensus motif are hydrophobic residues (I332/A333 in Amer1-A1, L505/Y506 in Amer1-A2, M372/A373 in Amer1-A4, L185/A186 in Asef-ABR, and T386/T387 in Sam68-YY), which are surrounded by non-polar APC residues such as F458, M503, and F510 (Supplementary Figure S10). Therefore, these APC-binding partners all possess a common consensus motif to be recognized by the same assembly of APC–ARM surface groove residues.

## Discussion

APC is a key regulatory molecule in the Wnt signaling pathway under both physiological and pathological conditions. It also has a crucial role in regulating the cytoskeleton formation and cell migration. The ARM domain of APC is critical for these diverse activities by mediating interactions with Amer1, Asef, IQGAP1, and so on to regulate these diverse functions. It is therefore of high appealing interest to determine the molecular basis of how APC–ARM accommodates these various binding partners. Intriguingly, although there is no apparent sequence similarity among the APC-binding regions A1, A2, and A4 of Amer1 and those of Asef and Sam68, our structural and mutational analysis revealed a common interaction mode shared by them and allowed us to deduce a consensus sequence for APC–ARM recognition (Figure 7c). We propose, and showed for the Asef and Amer1-A2 pair (Figure 7b), that these APC–ARM-binding partners are competitive with each other for APC-binding, similar to the case for β-catenin.

There are four APC-binding sites in Amer1, the previously described A1, A2, A3 [11] and the newly identified A4 in this study. These four sites are the most highly conserved regions of Amer1 (Figure 4d), implicating that the interaction between APC and Amer1 at these four sites is specially retained by the selection pressure throughout evolution [27]. Single-point mutations of key interface residues on APC or Amer1 attenuated their associations mediated through the four individual motifs (Figure 3), while composite mutations disrupting all four binding sites abrogated interactions of full-length proteins (Figure 5) and abolished the recruitment of APC to the plasma membrane by Amer1 (Figure 6).

By comparing the interaction modes of Amer1-A1, -A2, -A4, Asef-ABR, and Sam68-YY, a consensus APC–ARM-binding motif is proposed (Figure 7c). Side chain amide groups of four asparagine residues, N507/N550/N594/N641 from the H3 helices of APC armadillo repeats 2 through 4, hydrogen bond with the main-chain NH and CO groups of the APC–ARM-binding motif in a zipper-like manner. In addition, three glycines, one acidic residue, and two hydrophobic residues of the consensus APC–ARM-interacting motif are recognized by W553/W593, G511/K516, and F458/M503/F510 of APC, respectively (Supplementary Figure S10). It would be intriguing to investigate whether other APC–ARM-associating proteins such as IQGAP1 [15], PP2A-B56α [29], KAP3 [30], and Striatin [31] also use similar mechanisms to recognize APC. However, it would not be surprising if any of them employs a different recognition mode, as Amer1-A3 has already provided such a scenario.

Amer1 is a multifunctional protein whose gene knockout in mice resulted in defective specification of mesenchymal progenitor cell fate, mainly through aberrant β-catenin activation [17]. As the Amer1–APC complex is also involved in regulating β-catenin protein stability [19, 20], it would be worthwhile to investigate what effect specific disruption of the Amer1–APC complex would have on the Wnt/β-catenin signaling pathway. Both APC and Amer1 are scaffold proteins containing binding sites for both axin/conductin and β-catenin. Besides, there are four APC-binding sites in Amer1. Therefore, there might exist a gigantic multimeric APC–Amer1–axin–β-catenin complex inside the cell to collaboratively facilitate the degradation of β-catenin.

There are two paralogs of Amer1 in vertebrates, Amer2/FAM123A and Amer3/FAM123C [11, 27], which were suggested to be also involved in embryonic development [32]. Similar to Amer1, they possess the APC-binding sequences A1, A2, and A4 as well (Figures 1a and d). Amer2 was reported to collaborate with APC to function in neuroectodermal patterning through regulating Wnt/β-catenin signaling [33], microtubule stability, and cell migration [34]. In contrast, Amer3 was found to be a positive regulator of Wnt/β-catenin signaling [35]. It would be interesting to look into what tissue-specific cellular or developmental defects would result if complex formation of APC-Amer2 or APC-Amer3 is specifically disrupted.

## Materials and Methods

### Protein expression and purification

The cDNA of the armadillo repeat (ARM) domain (residues 407–751 and 407–775) of human APC were cloned into a pET28a-derived (Novagen, Madison, WI, USA) vector and overexpressed as an N-terminally His-tagged protein. The cDNA of the APC-binding fragments of human Amer1 (full A1: 280–364, core A1: 325–335, full A2: 400–531, core A2: 496–508, A3: 766–823, and A4: 365–375) were cloned into the pGEX4T1 vector (GE Healthcare, Little Chalfont, UK) to be expressed as N-terminally GST-tagged proteins. All proteins were over-expressed in the *E. coli* strain BL21(DE3), and purified by the Ni^2+^-NTA affinity chromatography (Qiagen, Hilden, Germany) or the GST affinity chromatography (Sigma, St Louis, MO, USA). After further purification by the Superdex200 gel filtration chromatography, the purified proteins were concentrated to 20 mg ml^−1^.

The peptides of Amer1-A1 (residues 325–335, LTGCGDIIAEQ), -A2 (residues 496–508, PRDSYSGDALYEF), and -A4 (residues 365–375, YQGGGEEMALP) were chemically synthesized with free amine and carboxylate ends, and purified by reverse phase HPLC (Appeptide Company, Shanghai, China). The APC/Amer1-A1, APC/Amer1-A2, APC/Amer1-A4 complexes were prepared by mixing concentrated APC–ARM proteins with the Amer1-A1, -A2, or -A4 peptides, respectively, with molar ratios of 1:1.5.

### Crystallization and structure determination

Crystallization experiments were performed at 14 °C by the hanging-drop vapor-diffusion method. Crystals of the APC–ARM/Amer1-A1 complex were grown in 0.1 M MES, pH 6.5, and 12% PEG 20 000. Crystals of the APC–ARM/Amer1-A2 complex were obtained at 25% ethyleneglycol only. The APC–ARM/Amer1-A4 complex was crystallized under the condition of 0.1 M sodium/potassium phosphate, pH 6.2, 0.2 M NaCl, and 10% PEG 8000. Crystal diffraction data sets were all collected at the beamline BL17U1 at Shanghai Synchrotron Radiation Facility (China), and processed using the HKL2000 software [36].

Crystals of the APC–ARM/Amer1-A1 complex belonged to the *P*2_1_2_1_2_1_ space group, with one set of the complex in each asymmetric unit. The structure was determined at 1.90 Å by the method of molecular replacement with the CCP4 program Phaser [37], using the structure of APC–ARM by itself (PDB code: 3NMW) [10] as the searching model. After model-building by Coot [38] and refinement by the CCP4 program REFMAC [39, 40], the final model has an *R*/*R*_free_ factor of 18.06%/22.13%. In the Ramachandran plot, 99.4 and 0.6% of residues are in the most favored and allowed regions, respectively.

Crystals of the APC–ARM/Amer1-A2 complex belonged to the *P*1 space group, with six sets of complexes in the asymmetry unit. The structure was determined at 2.10 Å using the same method as above. After refinement, the model has an *R*/*R*_free_ factor of 19.34%/21.27%. In the Ramachandran plot, 99.1 and 0.9% of residues are in the most favored and allowed regions, respectively.

Crystals of the APC–ARM/Amer1-A4 complex belonged to the *P*2_1_2_1_2_1_ space group, with one set of the complex in each asymmetry unit. The structure was determined at 1.70 Å using the same method as above. After refinement, the model has an *R*/*R*_free_ factor of 19.67%/23.98%. In the Ramachandran plot, 99.1 and 0.9% of residues are in the most favored and allowed regions, respectively.

The model qualities were all checked with the PROCHECK program [40].

### GST pull-down assays

GST pull-down assays between wild type (WT) or mutant APC–ARM proteins and WT/mutant Amer1 fragments were performed according to standard procedures as described previously [41]. The APC–ARM protein was added to pre-immobilized GST-Amer1 fragments on the GST affinity column at 4 °C, and then washed extensively using the GST column binding buffer (25 mM Tris-HCl, pH 8.0, 250 mM NaCl, 1 mM EDTA, and 14 mM β-mercaptoethanol). The bound proteins were eluted with the GST column elution buffer (50 mM Tris-HCl, pH 8.0, 300 mM NaCl, and 7 mM glutathione), and then analyzed by sodium dodecyl sulfate–polyacrylamide gel electrophoresis (SDS–PAGE) and Coomassie Blue staining.

### ITC assays

ITC experiments were performed using an ITC200 system (GE Healthcare) at 25 °C as described previously [42]. The buffer contained 50 mM HEPES, pH 7.5, 300 mM NaCl, and 1 mM EDTA. Typically, 200 μM APC–ARM protein was injected 20 times in 2 μl aliquots into a 200 μl sample cell containing GST-Amer1-A1/A2/A3/A4 protein at a concentration of 20 μM, or 1 mM GST-Amer1-A1/A2/A3/A4 protein was injected 20 times in 2 μl aliquots into a 200 μl sample cell containing APC–ARM protein at a concentration of 80 μM. Data were fit with a nonlinear least-square routine using a single-site binding model with Origin for ITC version 7.0 (MicroCal, Worcestershire, UK), varying the stoichiometry (*n*), the enthalpy of the reaction (Δ*H*), and the association constant (*K*_a_).

### *In vitro* binding competition assay

The competition assay between Asef-ABR-SH3 and Amer1-A2 for binding to APC–ARM was performed by the GST pull-down method. Four milliliters of the GST-Amer1-A2/APC–ARM complex, with the concentration of 0.5 mg ml^−1^, was preloaded onto the GST affinity column (1 ml) at 4 °C. 0.1 mg ml^−1^, 0.2 mg ml^−1^, 0.5 mg ml^−1^, and 1 mg ml^−1^ of the ABR-SH3 domain (residues 170–271) of Asef in a volume of 0.5 ml were then added to the reactions as competitors. After extensive washing by the GST column binding buffer, the bound proteins were eluted and analyzed by SDS–PAGE and Coomassie blue staining.

### Molecular graphics

All protein structure figures were generated with PyMOL (http://www.pymol.org).

### Cell culture and transfection

All cell lines were cultured in DMEM (PAA-Laboratories, Dartmouth, MA, USA) supplemented with 10% fetal calf serum (Perbio Science, Northumberland, UK) and 1% penicillin/streptomycin (PAA-Laboratories) at 37 °C in a humidified atmosphere of 10% CO_2_. Transient plasmid transfections were performed using polyethyleneimine (Sigma-Aldrich, St Louis, MO, USA) for HEK293T cells (3 μg of each plasmid) or TransIT-TKO (Mirus Bio LLC, Madison, WI, USA) for MCF-7 cells (1 μg of each plasmid).

### Plasmids

The following plasmids have been described previously: pCMV-APC [43], pBTM-APC–ARM (residues 308–789) and the N507K mutant [11], and pcDNA-Flag-Amer1 [11]. Flag- or EGFP-tagged Amer1 (2–700) and corresponding mutants were generated by PCR amplification and PCR mutagenesis, respectively, using human pEGFP-Amer1 as a template. The APC–ARM mutants were created by PCR mutagenesis and cloned into pBTM116 or pEGFP-C3 (Clonetech, Mountain View, CA, USA) expression vectors. The pEGFP-APC–ARM-K516E/M717K double mutant was generated by restriction digestion and re-ligation of each singly mutated construct. For yeast two-hybrid analysis the following constructs of Amer1 APC–ARM-binding sites cloned into pVP16 were used: Amer1-A1 (murine, residues 279–367), Amer1-A1 (human, residues 280–369), Amer1-A2 (murine, residues 388–551), Amer1-A2 (human, residues 380–531), Amer1-A3 (murine, residues 721–838) and Amer1-A4 (human, residues 337–455). Murine constructs have been described previously [11]. Human constructs were created by PCR amplification and mutants by PCR mutagenesis, respectively, using pEGFP-human Amer1 as a template.

### Preparation of protein lysates, immunoprecipitation, and western blotting

Immunoprecipitation experiments were performed in HEK293T cells. Protein lysates, immunoprecipitation experiments of GFP-tagged proteins, and western blotting were performed as described previously [18]. For immunoprecipitation of Flag-tagged proteins, lysates were incubated with anti-FLAG M2 affinity gel beads (Sigma-Aldrich). Bands in Figure 5d were quantified using Aida Image Analyzer Version 3.52.046 (Straubenhardt, Germany).

#### Immunofluorescence microscopy

Immunofluorescence experiments were performed in MCF-7 cells. Immunofluorescence staining and microscopy were performed as described previously [44].

#### Antibodies

Commercial antibodies were obtained from Abcam (Cambridge, UK) (mouse anti-APC, Ali), Roche (Basel, Switzerland) (mouse anti-GFP, mixture of clones 7.1 and 13.1), Serotec (Kidlington, UK) (rat anti-α-tubulin, clone YL1/2), Santa Cruz (Dallas, TX, USA) (mouse anti-VP16 and mouse anti-LexA) and Sigma (rabbit anti-Flag). Secondary antibodies coupled to horseradish peroxidase or Cy3 were purchased from Jackson ImmunoResearch (West Grove, PA, USA).

#### Yeast two-hybrid assay

Yeast two-hybrid and β-galactosidase assays were performed in the L40 yeast strain using pBTM116 as a bait vector and pVP16 as a prey vector as described previously [45] using 7 mM or 10 mM 3-Aminotriazole for background suppression of cell growth in Amer1-A4 or -A1, -A2, -A3 experiments, respectively.

### Accession codes

The atomic coordinates and structure factors of the APC–ARM/Amer1-A1, APC-ARM/Amer1-A2, and APC-ARM/Amer1-A4 complexes have been deposited in the Protein Data Bank with accession numbers of 4YJE, 4YJL, and 4YK6, respectively.

## Figures and Tables

**Table 1:** Dissociation constants (*K*_d_) between various Amer1 fragments and APC–ARM constructs as measured by the ITC assay

*Human Amer1/WTX*	*Human APC*	*Amer1/APC ratio*	*K*_*d*_ *(μM)*	*ΔH (kcal mol^−1^)*	*TΔS (kcal mol^−1^)*
Amer1 (280–368)	APC (407–751)	0.97±0.39	8.0±4.1	−1.12±0.55	5.8
Amer1 (325–335)	APC (407–751)	0.90±0.08	14.4±4.3	−0.90±0.10	5.7
Amer1 (325–335)	APC (407–751) N507K	NA	NA	NA	NA
Amer1 (325–335)	APC (407–751) K516E	NA	NA	NA	NA
Amer1 (380–531)	APC (407–751)	0.92±0.02	0.5±0.1	−3.5±0.1	5.1
Amer1 (496–508)	APC (407–751)	1.08±0.02	1.1±0.2	−5.2±0.2	2.9
Amer1 (496–508)	APC (407–751) N507K	NA	NA	NA	NA
Amer1 (496–508)	APC (407–751) K516E	NA	NA	NA	NA
Amer1 (496–508) D503A	APC (407–751)	NA	NA	NA	NA
Amer1 (496–508) L505D	APC (407–751)	NA	NA	NA	NA
Amer1 (716–834)	APC (407–775)	0.99±0.02	0.13±0.06	−1.82±0.06	7.6
Amer1 (766–823)	APC (407–775)	1.00±0.02	0.27±0.07	−1.82±0.04	7.2
Amer1 (766–823, Δ778–794)	APC (407–775)	1.02±0.03	0.22±0.11	−1.39±0.06	7.7
Amer1 (766–777)	APC (407–775)	NA	NA	NA	NA
Amer1 (786–823)	APC (407–775)	NA	NA	NA	NA
Amer1 (365–375)	APC (407–751)	0.95±0.02	2.0±0.3	−3.5±0.1	4.3

Abbreviations: APC, Adenomatous polyposis coli; Amer1, APC membrane recruitment 1; ITC, isothermal titration calorimetry.

‘NA’ refers to that no detectable interaction was observed.

**Table 2:** Data collection and refinement statistics

	*APC–ARM/Amer1-A1*	*APC–ARM/Amer1-A2*	*APC–ARM/Amer1-A4*
*Data collection*			
Space group	*P*2_1_2_1_2_1_	*P*1	*P*2_1_2_1_2_1_
Wavelength (Å)	0.97935	0.97935	0.97935
Unit-cell parameters: a, b, c (Å); α, β, γ (°)	53.2, 68.2, 93.4; 90, 90, 90	60.6, 168.9, 120.3; 60.3, 90.1, 90.1	49.4, 71.2, 91.7; 90, 90, 90
Number of molecules/asymmetric unit	1	6	1
Resolution range (Å)	50–1.90 (1.97–1.90)	50–2.10 (2.18–2.10)	50–1.70 (1.76–1.70)
Completeness (%)	99.9 (99.9)	98.3 (97.3)	99.6 (99.4)
Redundancy	13.4 (13.6)	3.9 (3.9)	14.5 (14.8)
Total observations	366,645	1,321,122	526,511
Unique reflections	27,310	335,892	36,291
*R*_merge_ (%)	9.7 (36.6)	10.3 (53.8)	7.7 (40.1)
I/*σ*_I_	22.8 (9.1)	13.4 (3.0)	30.4 (7.6)
CC1/2		0.858	0.978
			
*Refinement*			
*R*_work_ (%)	18.06	19.34	19.67
*R*_free_ (%)	22.13	21.27	23.98
Overall B factor	22.31	26.87	25.43
RMSD bond lengths (Å)	0.008	0.008	0.012
RMSD bond angles (°)	1.010	0.986	1.515
Ramanchandran plot (favored, allowed, disallowed, % )	99.4, 0.6, 0	99.1, 0.9, 0	99.1, 0.9, 0
Final model (number of protein/water atoms)	2 662/271	16 469/2 515	2 734/175

*R*_merge_=Σ*_h_*Σ*_i_* |*I*_*h*,*i*_−*I_h_*|/Σ*_h_*Σ*_i_* *I*_*h*,*i*_ for the intensity (*I*) of observation *i* of reflection *h*. *R* factor=Σ||*F*_obs_|−|*F*_calc_||/Σ|*F*_obs_|, where *F*_obs_ and *F*_calc_ are the observed and calculated structure factors, respectively. *R*_free_=*R* factor calculated using 5% of the reflection data chosen randomly and omitted from the start of refinement. RMSD, root-mean-square deviations from ideal geometry. Data for the highest resolution shell are shown in parentheses.

## References

[bib1] MacDonald BT, Tamai K, He X. Wnt/β-catenin signaling: components, mechanisms, and diseases. Dev Cell 2009; 17: 9–26. 19619488 10.1016/j.devcel.2009.06.016 PMC2861485

[bib2] Hamada F, Bienz M. A *Drosophila* APC tumor suppressor homologue functions in cellular adhesion. Nat Cell Biol 2002; 4: 208–213. 11862214 10.1038/ncb755

[bib3] Bienz M, Hamada F. Adenomatous polyposis coli proteins and cell adhesion. Curr Opin Cell Biol 2004; 16: 528–535. 15363803 10.1016/j.ceb.2004.08.001

[bib4] Akiyama T, Kawasaki Y. Wnt signalling and the actin cytoskeleton. Oncogene 2006; 25: 7538–7544. 17143298 10.1038/sj.onc.1210063

[bib5] Etienne-Manneville S. APC in cell migration. Adv Exp Med Biol 2009; 656: 30–40. 19928350 10.1007/978-1-4419-1145-2_3

[bib6] Caldwell CM, Kaplan KB. The role of APC in mitosis and in chromosome instability. Adv Exp Med Biol 2009; 656: 51–64. 19928352 10.1007/978-1-4419-1145-2_5

[bib7] Kinzler KW, Vogelstein B. Lessons from hereditary colorectal cancer. Cell 1996; 87: 159–170. 8861899 10.1016/s0092-8674(00)81333-1

[bib8] Bienz M, Clevers H. Linking colorectal cancer to Wnt signaling. Cell 2000; 103: 311–320. 11057903 10.1016/s0092-8674(00)00122-7

[bib9] Zhang Z, Lin K, Gao L, Chen L, Shi X, Wu G. Crystal structure of the armadillo repeat domain of adenomatous polyposis coli which reveals its inherent flexibility. Biochem Biophys Res Commun 2011; 412: 732–736. 21871439 10.1016/j.bbrc.2011.08.044

[bib10] Zhang Z, Chen L, Gao L et al. Structural basis for the recognition of Asef by adenomatous polyposis coli. Cell Res 2012; 22: 372–386. 21788986 10.1038/cr.2011.119 PMC3271586

[bib11] Grohmann A, Tanneberger K, Alzner A, Schneikert J, Behrens J. AMER1 regulates the distribution of the tumor suppressor APC between microtubules and the plasma membrane. J. Cell Sci 2007; 120: 3738–3747. 17925383 10.1242/jcs.011320

[bib12] Rivera MN, Kim WJ, Wells J et al. An X chromosome gene, *WTX*, is commonly inactivated in Wilms tumor. Science 2007; 315: 642–645. 17204608 10.1126/science.1137509

[bib13] Kawasaki Y, Senda T, Ishidate T et al. Asef, a link between the tumor suppressor APC and G-protein signaling. Science 2000; 289: 1194–1197. 10947987 10.1126/science.289.5482.1194

[bib14] Morishita EC, Murayama K, Kato-Murayama M et al. Crystal structure of the armadillo repeat domain of Adenomatous Polyposis Coli and its complex with the tyrosine-rich domain of Sam68. Structure 2011; 19: 1496–1508. 22000517 10.1016/j.str.2011.07.013

[bib15] Watanabe T, Wang S, Noritake J et al. Interaction with IQGAP1 links APC to Rac1, Cdc42, and actin filaments during cell polarization and migration. Dev Cell 2004; 7: 871–883. 15572129 10.1016/j.devcel.2004.10.017

[bib16] Jenkins ZA, van Kogelenberg M, Morgan T et al. Germline mutations in *WTX* cause a sclerosing skeletal dysplasia but do not predispose to tumorigenesis. Nat Genet 2009; 41: 95–100. 19079258 10.1038/ng.270

[bib17] Moisan A, Rivera MN, Lotinun S et al. The *WTX* tumor suppressor regulates mesenchymal progenitor cell fate specification. Dev Cell 2011; 20: 583–596. 21571217 10.1016/j.devcel.2011.03.013 PMC4052985

[bib18] Tanneberger K, Pfister AS, Kriz V, Bryja V, Schambony A, Behrens J. Structural and functional characterization of the Wnt inhibitor APC membrane recruitment 1 (Amer1). J Biol Chem 2011; 286: 19204–19214. 21498506 10.1074/jbc.M111.224881 PMC3103299

[bib19] Major MB, Camp ND, Berndt JD et al. Wilms tumor suppressor WTX negatively regulates Wnt/β-catenin signaling. Science 2007; 316: 1043–1046. 17510365 10.1126/science/1141515

[bib20] Tanneberger K, Pfister AS, Brauburger K et al. Amer1/WTX couples Wnt-induced formation of PtdIns(4,5)P_2_ to LRP6 phosphorylation. EMBO J 2011; 30: 1433–1443. 21304492 10.1038/emboj.2011.28 PMC3102280

[bib21] Rivera MN, Kim WJ, Wells J et al. The tumor suppressor WTX shuttles to the nucleus and modulates WT1 activity. Proc Natl Acad Sci USA 2009; 106: 8338–8343. 19416806 10.1073/pnas.0811349106 PMC2677091

[bib22] Kim WJ, Rivera MN, Coffman EJ, Haber DA. The WTX tumor suppressor enhances p53 acetylation by CBP/p300. Mol Cell 2012; 45: 587–597. 22285752 10.1016/j.molcel.2011.12.025 PMC3310179

[bib23] Ha NC, Tonozuka T, Stamos JL, Choi HJ, Weis WI. Mechanism of phosphorylation-dependent binding of APC to β-catenin and its role in β-catenin degradation. Mol Cell 2004; 15: 511–521. 15327768 10.1016/j.molcel.2004.08.010

[bib24] Xing Y, Clements WK, Le Trong I et al. Crystal structure of a β-catenin/APC complex reveals a critical role for APC phosphorylation in APC function. Mol Cell 2004; 15: 523–533. 15327769 10.1016/j.molcel.2004.08.001

[bib25] Huber AH, Weis WI. The structure of the β-catenin/E-cadherin complex and the molecular basis of diverse ligand recognition by β-catenin. Cell 2001; 105: 391–402. 11348595 10.1016/s0092-8674(01)00330-0

[bib26] Graham TA, Weaver C, Mao F, Kimelman D, Xu W. Crystal structure of a β-catenin/Tcf complex. Cell 2000; 103: 885–896. 11136974 10.1016/s0092-8674(00)00192-6

[bib27] Boutet A, Comai G, Schedl A. The *WTX/AMER1* gene family: evolution, signature, and function. BMC Evol Biol 2010; 10: 280. 20843316 10.1186/1471-2148-10-280 PMC2949870

[bib28] Mitin N, Betts L, Yohe ME, Der CJ, Sondek J, Rossman KL. Release of autoinhibition of ASEF by APC leads to CDC42 activation and tumor suppression. Nat Struct Mol Biol 2007; 14: 814–823. 17704816 10.1038/nsmb1290 PMC2716141

[bib29] Seeling JM, Miller JR, Gil R, Moon RT, White R, Virshup DM. Regulation of β-catenin signaling by the B56 subunit of protein phosphatase 2A. Science 1999; 283: 2089–2091. 10092233 10.1126/science.283.5410.2089

[bib30] Jimbo T, Kawasaki Y, Koyama R et al. Identification of a link between the tumor suppressor APC and the kinesin superfamily. Nat Cell Biol 2002; 4: 323–327. 11912492 10.1038/ncb779

[bib31] Breitman M, Zilberberg A, Caspi M, Rosin-Arbesfeld R. The armadillo repeat domain of the APC tumour suppressor protein interacts with Striatin family members. Biochim Biophys Acta 2008; 1783: 1792–1802. 18502210 10.1016/j.bbamcr.2008.04.017

[bib32] Comai G, Boutet A, Neirijnck Y, Schedl A. Expression patterns of the *Wtx/Amer* gene family during mouse embryonic development. Dev Dyn 2010; 239: 1867–1878. 20503382 10.1002/dvdy.22313

[bib33] Pfister AS, Tanneberger K, Schambony A, Behrens J. Amer2 is a novel negative regulator of Wnt/β-catenin signaling involved in neuroectodermal patterning. J Biol Chem 2012; 287: 1734–1741. 22128170 10.1074/jbc.M111.308650 PMC3265856

[bib34] Pfister AS, Hadjihannas MV, Röhrig W, Schambony A, Behrens J. Amer2 interacts with EB1 and APC and controls microtubule stability and cell migration. J Biol Chem 2012; 287: 35333–35340. 22898821 10.1074/jbc.M112.385393 PMC3471750

[bib35] Brauburger K, Akyildiz S, Ruppert JG et al. Adenomatous polyposis coli (APC) membrane recruitment 3, a member of the APC membrane recruitment family of APC-binding proteins, is a positive regulator of Wnt-β-catenin signalling. FEBS J 2014; 281: 787–801. 24251807 10.1111/febs.12624

[bib36] Otwinowski Z, Minor W. Methods Enzymol. Academic Press:New York, 1997; 276. 10.1016/S0076-6879(97)76066-X 27754618

[bib37] McCoy AJ, Grosse-Kunstleve RW, Adams PD, Winn MD, Storoni LC, Read RJ. Phaser crystallographic software. J Appl Crystallogr 2007; 40: 658–674. 19461840 10.1107/S0021889807021206 PMC2483472

[bib38] Emsley P, Cowtan K. Coot: model-building tools for molecular graphics. Acta Crystallogr D 2004; 60: 2126–2132. 15572765 10.1107/S0907444904019158

[bib39] Winn MD, Murshudov GN, Papiz MZ. Macromolecular TLS refinement in REFMAC at moderate resolutions. Methods Enzymol 2003; 374: 300–321. 14696379 10.1016/S0076-6879(03)74014-2

[bib40] Collaborative Computational Project Number 4. The CCP4 suite: programs for protein crystallography. Acta Crystallogr D 1994; 50: 760–763. 15299374 10.1107/S0907444994003112

[bib41] Zhang Z, Li H, Chen L et al. Molecular basis for the recognition of adenomatous polyposis coli by the Discs Large 1 protein. PLoS ONE 2011; 6: e23507. 21858148 10.1371/journal.pone.0023507 PMC3157396

[bib42] Zhang Y, Fu L, Qi X et al. Structural insight into the mutual recognition and regulation between suppressor of fused and Gli/Ci. Nat Commun 2013; 4: 2608. 24217340 10.1038/ncomms3608 PMC5842692

[bib43] Smith KJ, Levy DB, Maupin P, Pollard TD, Vogelstein B, Kinzler KW. Wild-type but not mutant APC associates with the microtubule cytoskeleton. Cancer Res 1994; 54: 3672–3675. 8033082

[bib44] Behrens J, von Kries JP, Kühl M et al. Functional interaction of beta-catenin with the transcription factor LEF-1. Nature 1996; 382: 638–642. 8757136 10.1038/382638a0

[bib45] Behrens J, Jerchow BA, Würtele M et al. Functional interaction of an axin homolog, conductin, with beta-catenin, APC, and GSK3beta. Science 1998; 280: 596–599. 9554852 10.1126/science.280.5363.596

