# Supplementary material for: Structures of the APC–ARM domain in complexes with discrete Amer1/WTX fragments reveal that it uses a consensus mode to recognize its binding partners
Source: Cell Discov. 2015 Jul 14;1:15016–. doi: 10.1038/celldisc.2015.16 (PMC4860839; doi:10.1038/celldisc.2015.16)
Supplement: Supplementary Information [file celldisc201516-s1.doc]

**Supplementary Information**

**Supplementary Figures**

**
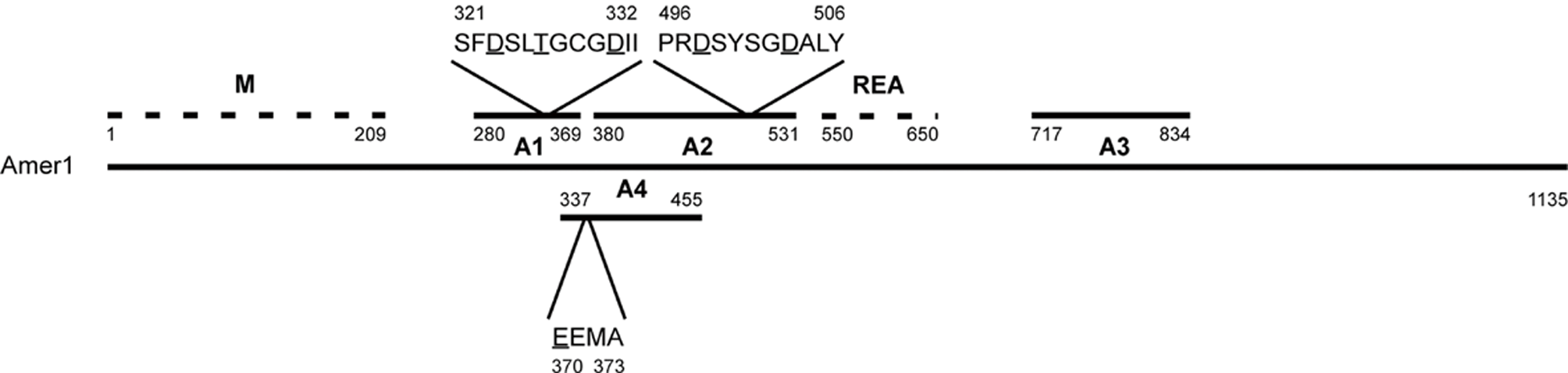
**

**Figure S1** Schematic representation of the human Amer1/WTX protein. The A1, A2, and A3 binding regions for APC as well as the newly discovered APC-binding site A4 are indicated. Highly conserved amino acids are shown. Residues mutated in the yeast two-hybrid assays are underlined. “M” in the figure stands for the N-terminal membrane localization region. The β-catenin-binding REA repeat region is indicated by dashed lines.


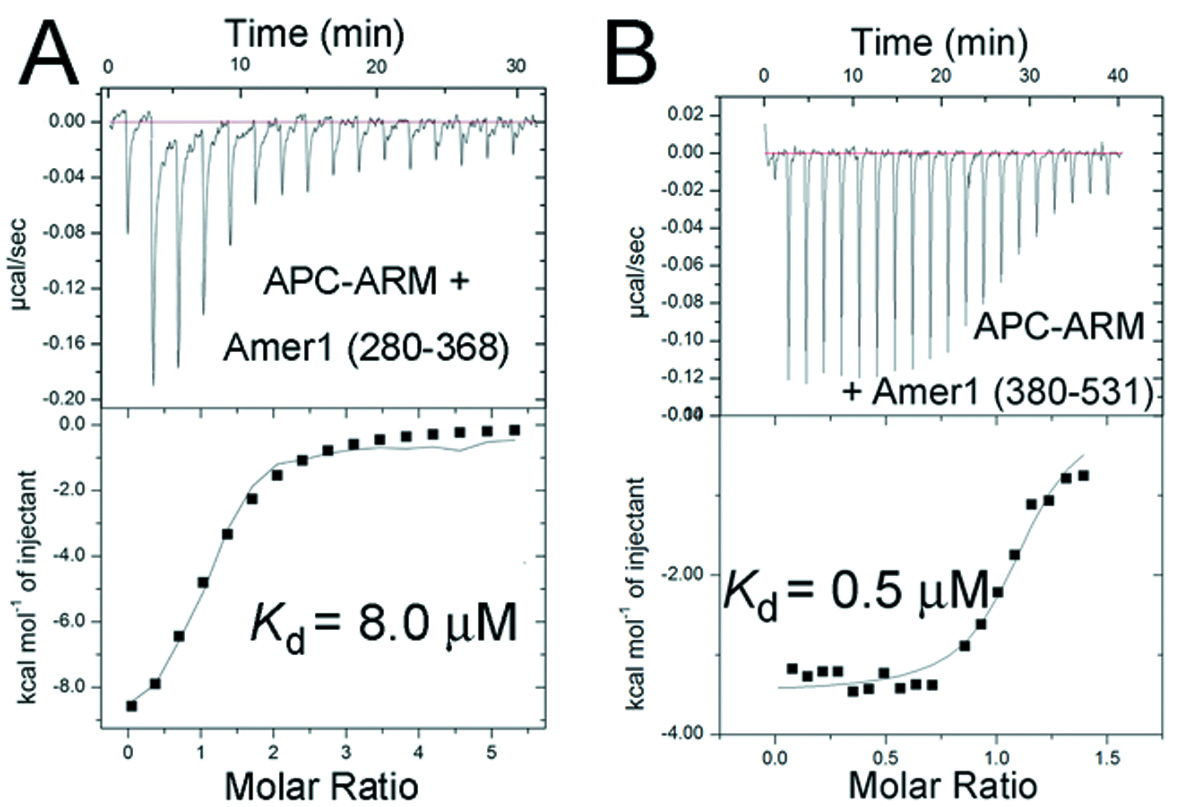


**Figure S2** Measurement of the dissociation constant (*K*_d_) values of Amer1-A1/A2 fragments for APC-ARM. **(A)** The dissociation constant (*K*_d_) of the full Amer1-A1 fragment (residues 280-368) for APC-ARM as measured by the ITC assay. **(B)** The dissociation constant (*K*_d_) of the full Amer1-A2 fragment (residues 380-531) for APC-ARM as measured by the ITC assay.


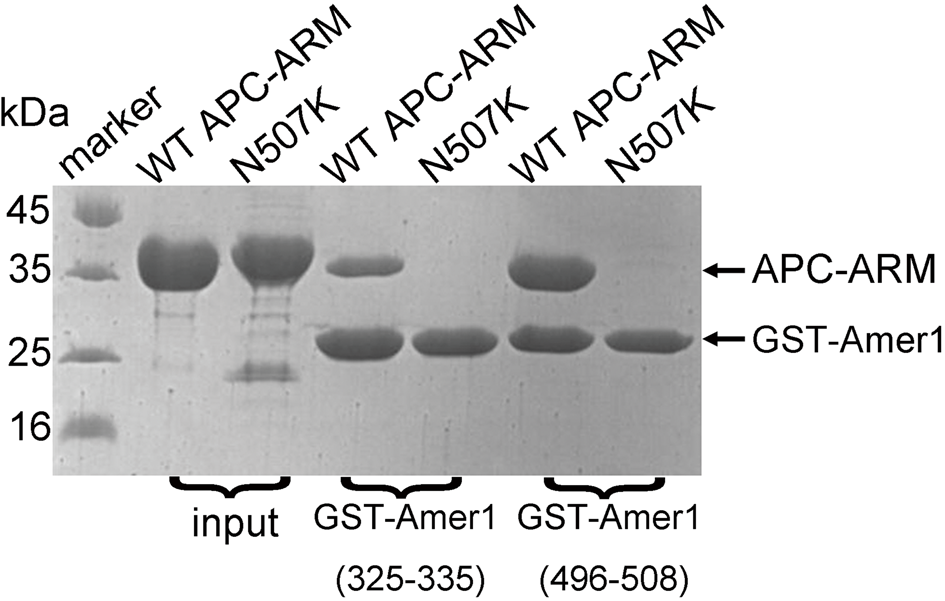


**Figure S3** In vitro GST pull-down binding assay between APC-ARM and GST-tagged Amer1-A1 (residues 325-335) or Amer1-A2 (residues 496-508). Both the two fragments of Amer1 displayed stable interactions with wild-type (WT) APC-ARM, but not with its N507K point mutant.


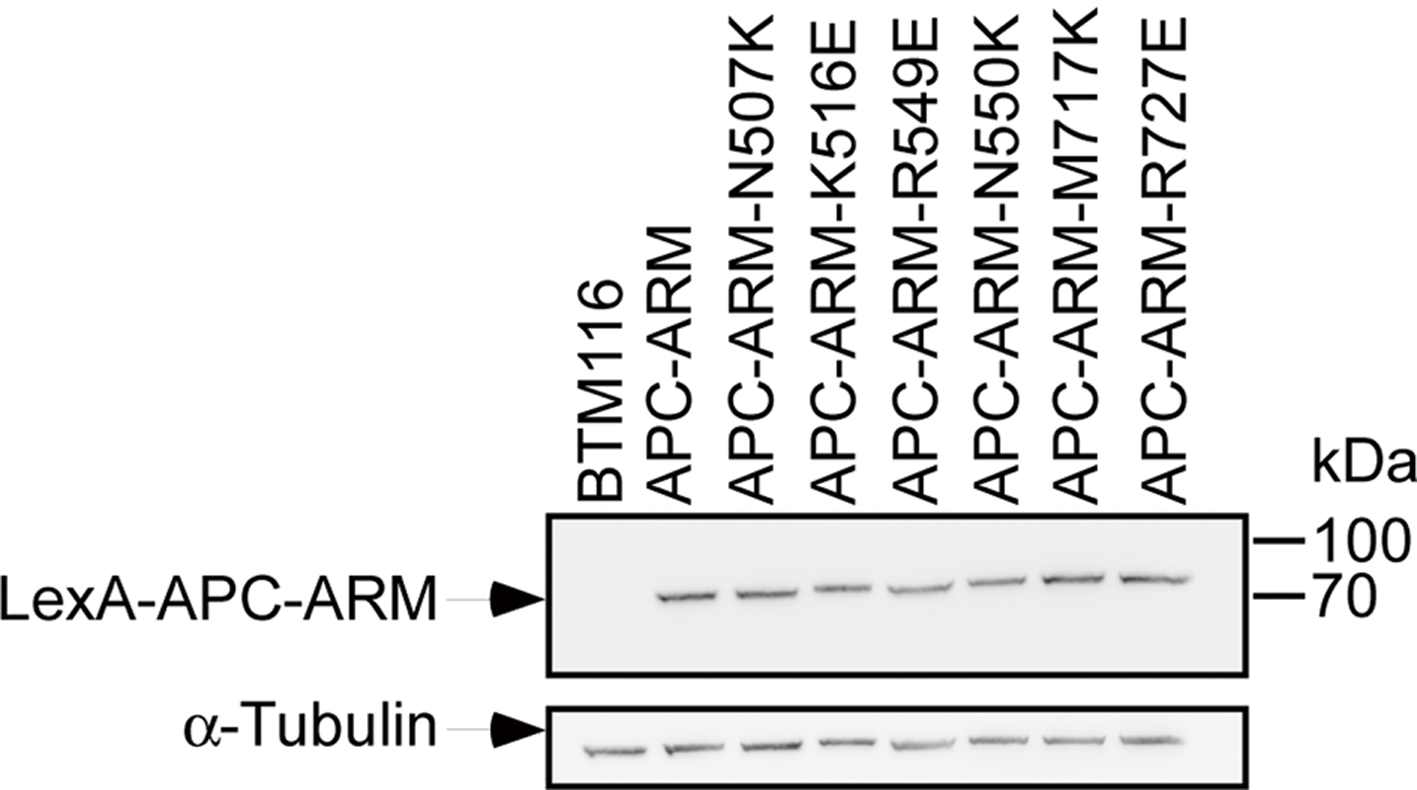


**Figure S4** Protein expression levels of WT and various point mutants of APC-ARM constructs used in the yeast two-hybrid assay.


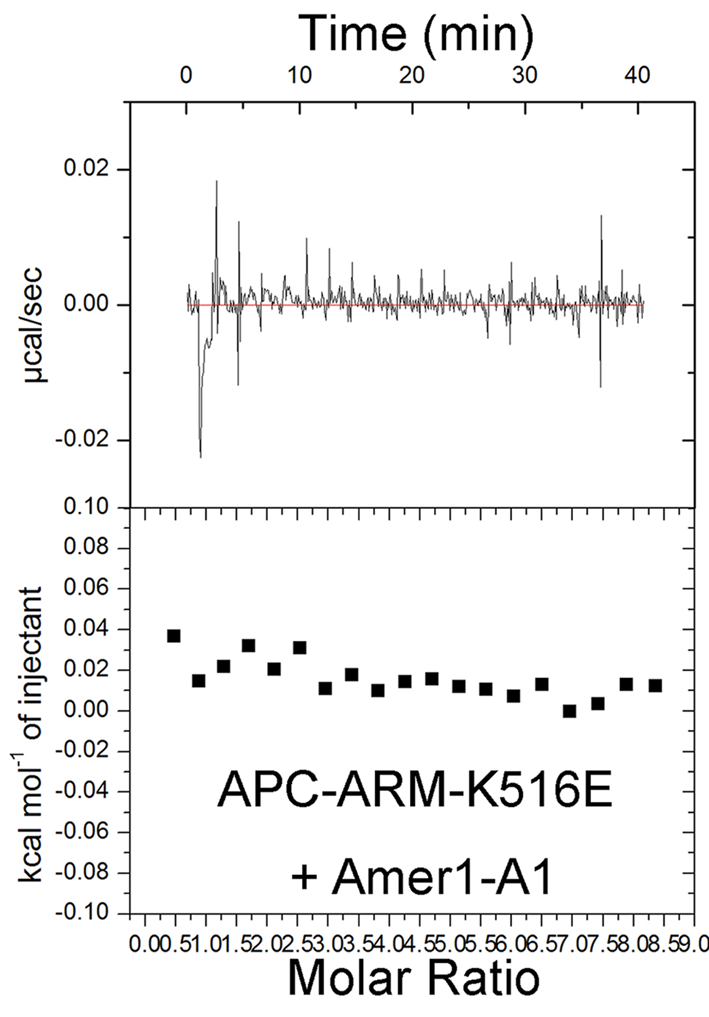


**Figure S5** The K516E point mutant of APC-ARM had non-detectable interaction with Amer1-A1, as measured by the ITC assay.


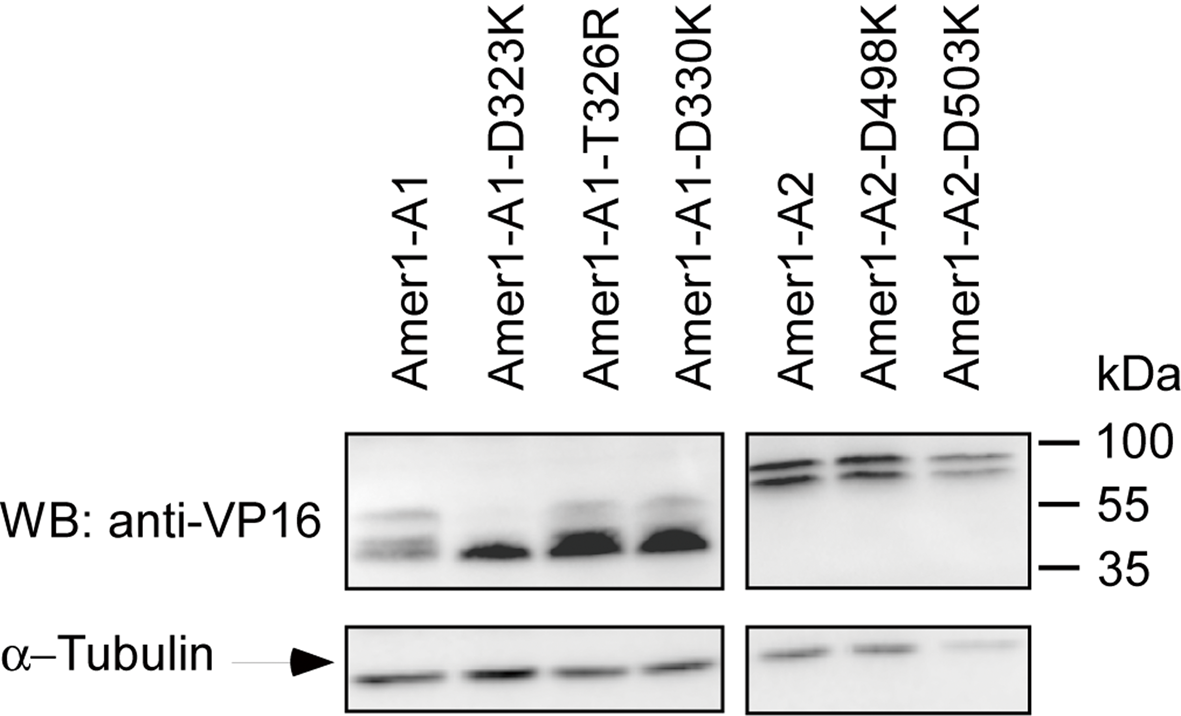


**Figure S6** Protein expression levels of WT human Amer1-A1 and its point mutants D323K, T326R, and D330K as well as WT human Amer1-A2 and its point mutants D498K and D503K which were used in the yeast two-hybrid assay.


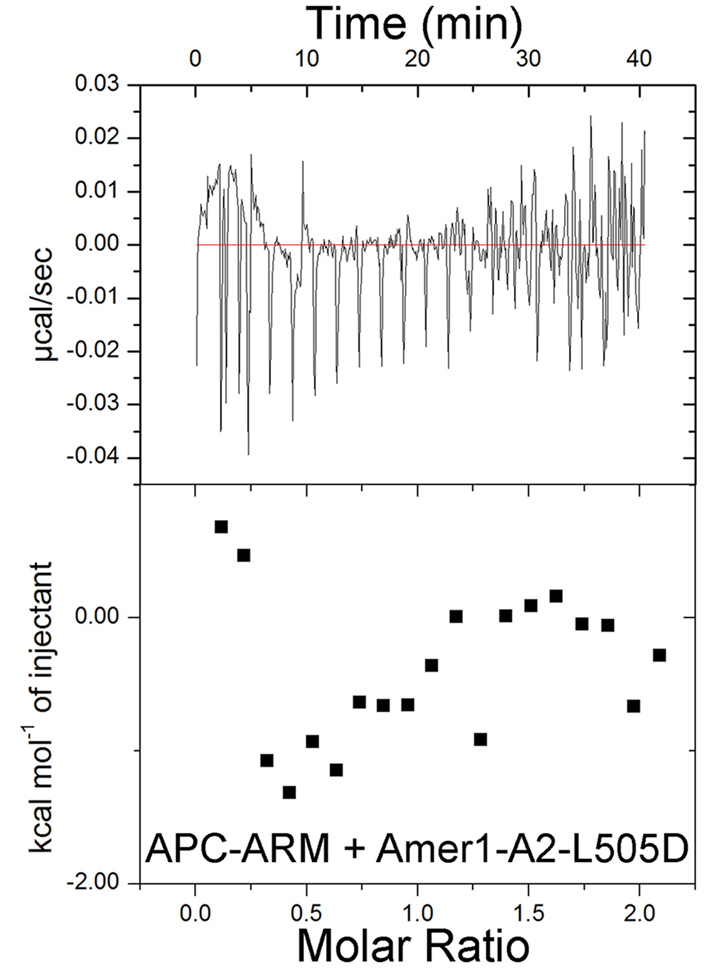


**Figure S7** The point mutation of L505D on Amer1-A2 abolished its recognition with APC-ARM, as manifested by the ITC assay.


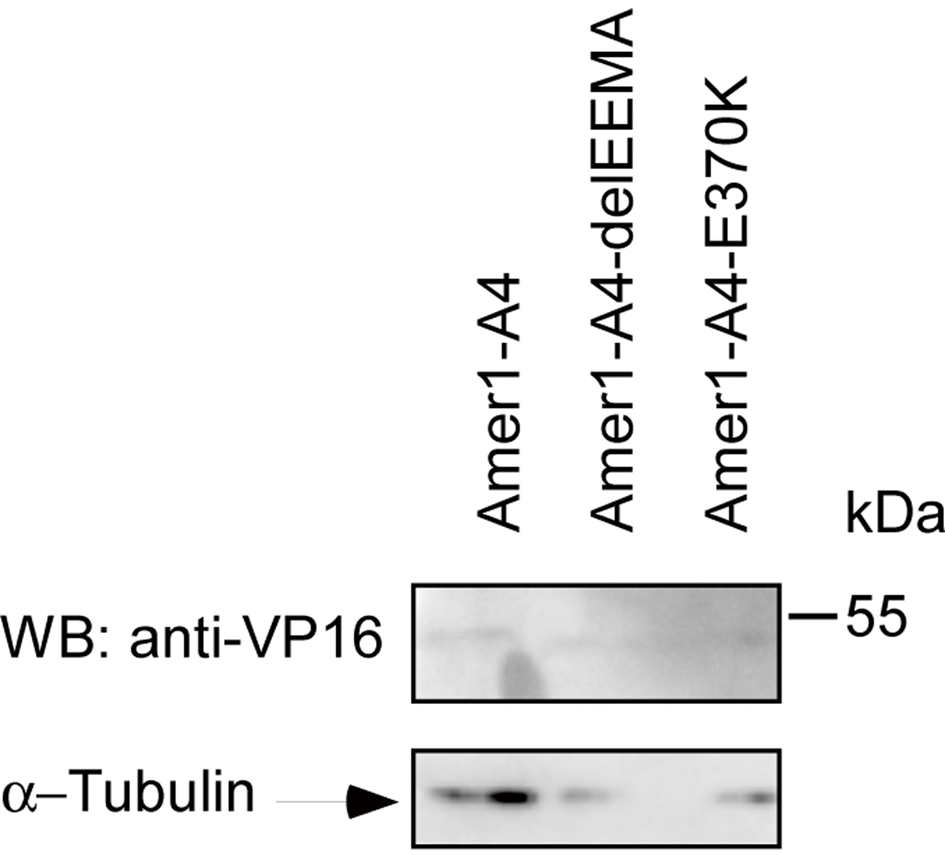


**Figure S8** Protein expression levels of WT human Amer1-A4, its point mutant E370K, as well as a deletion construct in which residues 370-373 were deleted (delEEMA) which were used in the yeast two-hybrid assay.


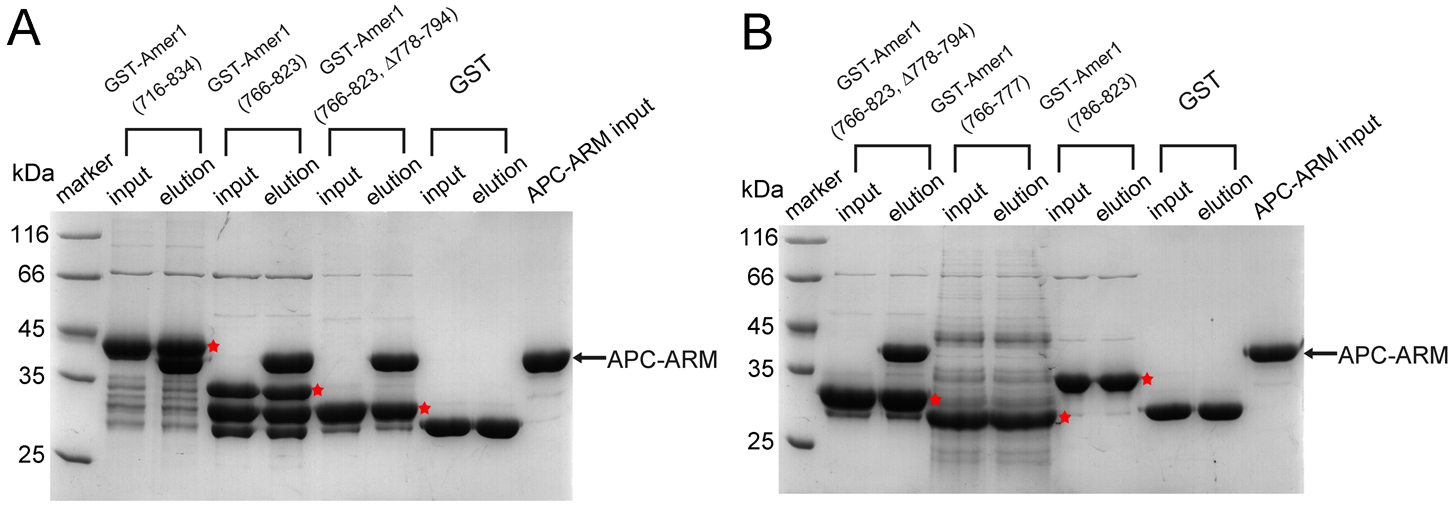


**Figure S9** Using the GST pull-down assay to search for the minimal binding fragment of Amer1-A3 for the association with APC-ARM. **(A)** Amer1 (766-823) and Amer1 (766-823, Δ778-794) interacted with APC-ARM as strongly as Amer1 (716-834), as demonstrated by the GST pull-down assay. GST-tagged Amer1 (716-834), Amer1 (766-823), and Amer1 (766-823, Δ778-794) proteins are marked by red asterisks. **(B)** Amer1 (766-777) and Amer1 (786-823) did not interact with APC-ARM, as shown by the GST pull-down assay. GST-tagged Amer1 (766-823, Δ778-794), Amer1 (766-777), and Amer1 (786-823) proteins are marked by red asterisks.


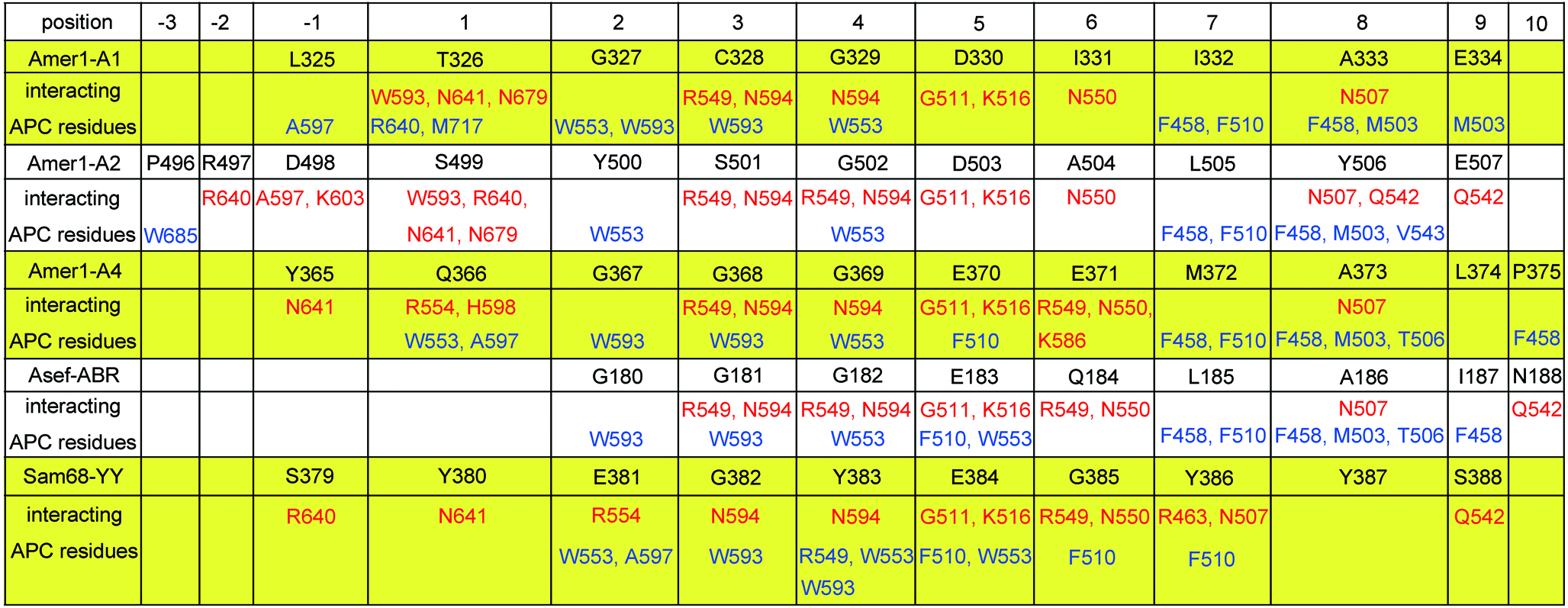


**Figure S10** Tabulation of the interaction details between APC-ARM and its binding partners Amer1-A1, -A2, -A4, Asef-ABR, and Sam68-YY. APC residues using hydrogen bonds or hydrophobic interactions to recognize its binding partners are colored in red and blue, respectively.

**Supplementary Tables**

**Table S1** Dihedral angles of the residues corresponding to the second, third, and fourth positions of the consensus motif in Amer1-A1, -A2, and -A4 peptides. Values for the glycine residues at the fourth position, which adopt special dihedral angles disallowed on the Ramachandran plot for the other nineteen kinds of amino acids, are shown in bold.

| Amer1-A1 | | | Amer1-A2 | | | Amer1-A4 | | |
| --- | --- | --- | --- | --- | --- | --- | --- | --- |
|  | φ | ψ |  | φ | ψ |  | φ | ψ |
| G327 | -161.134 | 177.693 | Y500 | -141.628 | 156.320 | G367 | -92.600 | -2.141 |
| C328 | -130.406 | 14.527 | S501 | -90.435 | -15.589 | G368 | -68.283 | -27.840 |
| **G329** | **70.370** | **-157.889** | **G502** | **78.470** | **-150.670** | **G369** | **70.835** | **-164.613** |
